# A pictorial review of acute aortic syndrome: discriminating and overlapping features as revealed by ECG-gated multidetector-row CT angiography

**DOI:** 10.1007/s13244-012-0195-7

**Published:** 2012-11-06

**Authors:** Takuya Ueda, Anne Chin, Ivan Petrovitch, Dominik Fleischmann

**Affiliations:** 1 Department of Radiology, St. Luke’s International Hospital, 9-1 Akashi-cho, Chuo-ku, Tokyo 104-8560 Japan; 2 Department of Radiology, Centre Hospitalier de L’Univeristé de Montréal, 3480 rue St. Urbain, Montreal, Quebec Canada; 3 Northern Virginia Radiology Consultants, PLLC, 1701 N. George Mason Drive, Arlington, VA 22205 USA; 4 Department of Radiology, Stanford University School of Medicine, 300 Pasteur Drive, Stanford, CA 94305 USA

**Keywords:** Aortic diseases, Tomography, X-ray computed, Aneurysm, Aortic dissection, Aortic aneurysm

## Abstract

**Background:**

The term "acute aortic syndrome" (AAS) encompasses a spectrum of life-threatening conditions characterized by acute aortic pain. AAS traditionally embraces three abnormalities including classic aortic dissection, intramural haematoma, and penetrating atherosclerotic ulcer. Although the underlying etiologies and conditions predisposing to AAS are diverse, the clinical features are indistinguishable.

**Methods:**

Multidetector-row computed tomography (CT) with electrocardiographic gating (ECG-gated MDCT) has greatly improved imaging of acute thoracic aortic diseases by virtually eliminating pulsation artifacts transmitted from cardiac motion and reveals subtle aortic abnormalities, which have been difficult to recognize by conventional non-gated CT.

**Results:**

While these advances in imaging technology provide additional discriminating features of acute aortic diseases, they also reveal a range of overlapping features of these life-threatening conditions that not uncommonly are dynamic and evolving. These overlapping and transitional features may be a major source of misunderstanding, confusion, and controversy for diseases that cause AAS.

**Conclusion:**

In this pictorial review, we describe the discriminating and typical imaging features as revealed by modern ECG-gated MDCT angiography. In addition to the discriminating features, recognition of the overlapping and transitional features in AAS will allow a more comprehensive understanding of their underlying pathophysiologic conditions and their natural history, and may improve therapeutic management.

**Main Messages:**

• *The superior visualization of ECG-gated CTA improves the diagnostic accuracy of acute aortic syndrome.*

• *ECG-gated CTA provides discriminating features of underlying pathophysiologic conditions of AAS.*

• *Also, recognition of the overlapping features in AAS will allow a more comprehensive understanding.*

## Introduction

### Acute aortic syndrome

The term ‘acute aortic syndrome’ (AAS) refers to a spectrum of acute, life-threatening conditions of aortic diseases characterized clinically by abrupt, intense chest and/or back pain [1]. AAS traditionally embraces three aortic diseases: aortic dissection (AD), intramural haematoma (IMH) and penetrating atherosclerotic ulcer (PAU) [1, 2]. The differentiation of disease entities in AAS is essential for predicting the natural history and for initiating the appropriate treatment in a timely fashion [2, 3]. However, the presenting clinical signs and symptoms of AAS of any etiology are usually clinically indistinguishable [4]. Although typical cases demonstrate characteristic imaging features of each disease, imaging findings may also overlap between different entities, especially when the process is dynamic and evolving. These transitional and overlapping features of AAS, both clinical and pertaining to imaging findings, have led to several misconceptions and controversies concerning the disease concept of AAS.

**Fig. 1:**
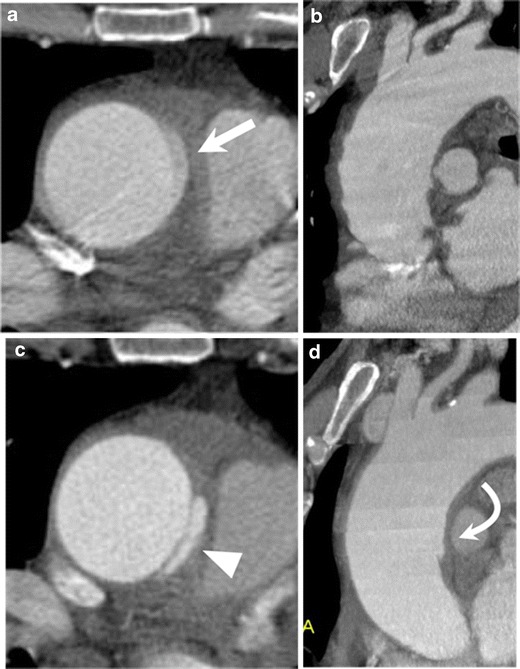
Impact of ECG-gated CT angiography (CTA). Non-gated (**a**, **b**) and ECG-gated (**c**, **d**) CTA in a 66-year-old man with limited intimal tear. **a** Non-gated axial CT image shows a ‘dissection-like’ motion artifact (*arrow*) on the contour of the aorta because of pulsatile motion of the aorta. **b** Oblique sagittal slab maximum intensity projection (slab-MIP) image shows an undulating contour of the aorta caused by misregistration from the pulsatile motion. **c** ECG-gated CTA, performed 1 day after the non-gated CT, eliminates the pulsatile motion artifact. The image clearly reveals a hidden focal intimal tear (*arrowhead*). **d** Slab MIP image of ECG-gated CTA demonstrates visualization of the ascending aorta without misregistration artifacts. A focal bulge with a tiny intimal tear is revealed (*curved arrow*)

CT angiography (CTA) is an established imaging technique to diagnose AAS in the emergency setting, and newer generation multidetector-row CT (MDCT) scanners have improved the sensitivities and specificities of aortic pathologies [5]. Additionally, the introduction of the retrospective or prospective electrocardiographic (ECG)-gated technique has virtually eliminated the notorious pulsation artifacts in the ascending aorta and allows for a substantially improved assessment of subtle aortic abnormalities previously under-recognized by non-gated CTA (Fig. 1) [6]. The superior visualization of ECG-gated CTA dramatically improves the detectability of some pathognomonic findings such as the primary intimal tear in thrombosed AD or ulcerative plaque in PAU [2, 6].

**Fig. 2:**
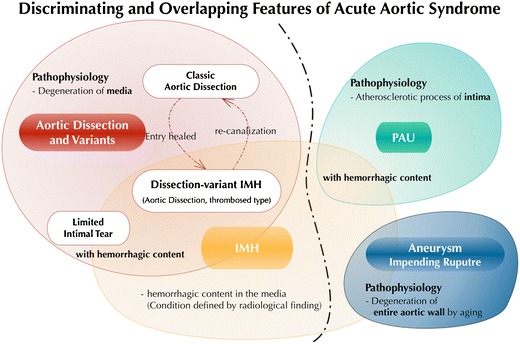
Discriminating and overlapping features of acute aortic syndrome. In addition to the certain discriminating features as revealed by recent advances in imaging, each disease in acute aortic syndrome (AAS) may show overlapping features and transition from one to another. In such overlapping and transitional features of aortic diseases, the diagnosis may be affected by the time point at which the imaging has been examined and also by the detectability of tiny pathology by the imaging modality. Awareness of some related and distinguishing radiologic features in AAS is helpful in understanding these diseases and providing new insight into the pathophysiology

In this pictorial review, we illustrate discriminating imaging features of AAS disease entities as well as the overlapping and transitional ones as revealed by modern ECG-gated CTA (Fig. 2). Awareness of some related and distinguishing radiologic features in AAS may improve our understanding of these diseases and provide further insight into the pathophysiology and natural history and guide appropriate management of these lesions.

## Aortic dissection

**Fig. 3:**
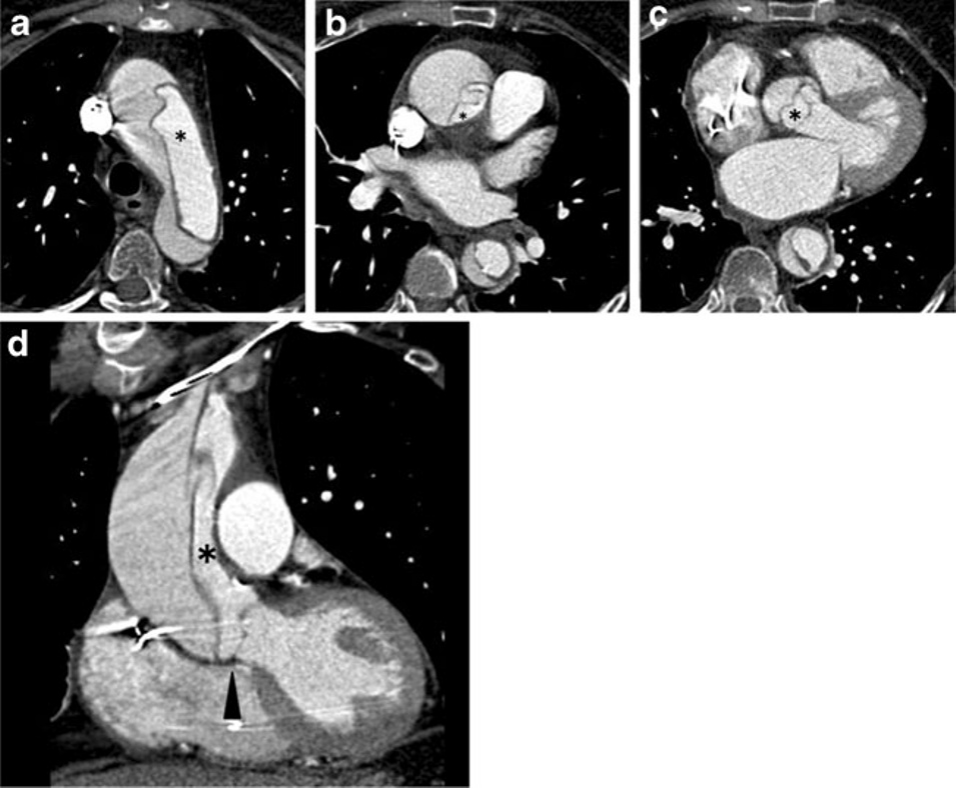
Acute Stanford type A classic aortic dissection in a 71-year-old woman with abrupt, severe retrosternal chest pain. **a**–**c** Transverse images from an ECG-gated CTA demonstrate an extensive intimo-medial flap involving the entire thoracic aorta. The true lumen (*) appears small in the ascending aorta where it is nearly circumferentially separated from the rest of the aortic wall (**b**). Extension of the flap down to the aortic root to involve the aortic valve apparatus is clearly demonstrated in this patient who had aortic regurgitation confirmed on echocardiography, a significant complication of acute dissection (**c**). **d** Multiplanar reformations in a coronal plane show the involvement of the brachiocephalic artery to better advantage (*arrow*), the near circumferential separation of the aortic media at the level of the ascending aorta and its extension down to the aortic valve apparatus (*arrowhead*)

Classic aortic dissection (AD) is characterized by separation of the layers of the aortic media initiated by a primary intimal tear, with variable longitudinal and circumferential extension, resulting in two distinct flow channels: the true lumen, contained entirely by the intima, and the false lumen, contained entirely within the medial layer of the aorta (Fig. 3) [7]. Degeneration of the medial layer of the aortic wall is the fundamental pathophysiology of AD [8]. The common pathologic denominator (process) resulting in medial degeneration, initially termed ‘cystic media necrosis’ by Erdheim in 1929, has histologic features characterized by non-inflammatory loss of vascular smooth muscle cells and elastolysis of aortic wall components [9]. ‘Cystic media necrosis’ is the common end path of various acquired and genetic conditions weakening the medial layers of the aorta leading to the breakdown in the integrity of the aortic wall, which precedes AD [10]. The most common comorbid condition for cystic media necrosis and AD is severe arterial hypertension. Chronic exposure of the aorta to high blood pressure may induce medial disruption and degeneration of the aortic wall [8]. In addition, several genetic conditions—such as Marfan syndrome, Ehlers-Danlos syndrome and familial aortic aneurysm/dissection—are well-known precursors of AD as well [11]. Intimal disease—namely atherosclerosis—is not a prerequisite for AD, although it may coexist particularly in the elderly. This is in contradistinction to PAU, which is essentially a manifestation of severe and advanced atherosclerosis [12].

Two anatomical systems—the DeBarkey and Stanford classification—are used to classify aortic dissection [2, 10]. Recently, the Stanford system has been more widely used worldwide as it is simpler and directly connected with the therapeutic strategy. Stanford type A dissection, which involves the ascending aorta regardless of the site of the primary intimal tear, should be treated as a surgical emergency as these patients are at high risk for a life-threatening complication with a high mortality rate (1–2 % per hour early after symptom onset) [2, 10]. Patients with uncomplicated Stanford type B dissection, which was confined to the aortic arch and the descending aorta, are best treated with medical therapy; the in-hospital mortality for these patients was 10% [2, 10]. Radiological imaging plays an essential role in determination of the therapeutic strategy.

### Discriminating and overlapping features of aortic dissection

Classic AD can be unambiguously differentiated from other forms of AAS by the presence of a double-barrel lumen, consisting of a true lumen and a false lumen, resulting from a primary intimal tear, which is usually clearly visualized with state-of-the-art CT technology: the false lumen is contained entirely within the aortic media and is separated from the true lumen by a dissection flap or ‘intimal flap.’ However, confusion with respect to the terminology arises when classic AD is associated with non-flowing blood or haemorrhagic content within the medial layer. This is not uncommon in situations when the primary intimal tear is more distal (e.g., in the descending aorta) than the most proximal extent of the medial separation (which may extend retrogradely into the ascending aorta), where stagnating blood or clots can be observed. In CT, stagnating blood in the false lumen is isodense to blood within the true lumen, whereas thrombosed blood is hyperdense relative to flowing blood in non-contrast CT. The extreme within the spectrum of separation of medial layers as a consequence of underlying ‘cystic media necrosis’ occurs in complete thrombosis of the false lumen, resulting in the imaging features of IMH, and in this context, IMH is considered a variant of classic AD. Another variant of AD—called limited intimal tear or discrete/subtle tear—is characterized by a complex primary intimal tear with only minimal separation between the medial layers at the edges of the lesion; this disease entity also has a pathophysiology and etiology overlapping with classic dissection [9, 13, 14]. These topics are elaborated below.

## Intramural haematoma

**Fig. 4:**
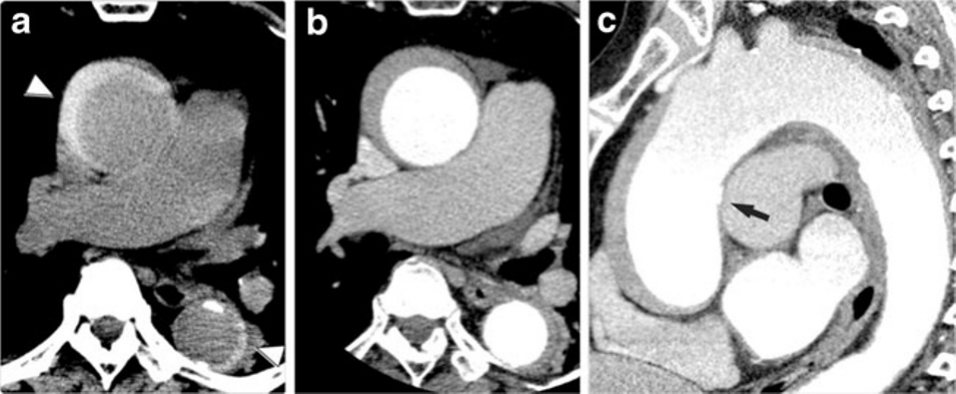
Dissection variant intramural haematoma (IMH) in a 54-year-old man who presented with acute chest pain. **a** Non-contrast CT image shows a crescent-shaped high-density area in the ascending aorta and descending aorta (*arrowhead*). **b** On the axial contrast-enhanced CT image, neither the flow channel nor intimal flap is detected along the entire aorta, consistent with IMH. **c** Oblique sagittal MPR image demonstrates widespread extension of type A IMH throughout the whole thoracic aorta. An ‘ulcer-like projection,’ the radiological finding of a focal disruption of the surface of the aortic wall from the true lumen into the thrombosed false lumen, is identified at the mid ascending aorta (*arrow*). During surgery, a small thrombosed focal primary intimal tear (PIT) was identified at the corresponding site

Intramural haematoma is defined as a hyperdense, crescent-shaped lesion within the aortic wall on non-enhanced CT (Fig. 4) [15, 16]. As defined originally, IMH has no demonstrable intimal flap or radiologically apparent intimal tear [17]. This view is outdated, however, since modern imaging technology can usually detect small communications between the true and false lumen [2].

The etiology of IMH remains controversial [17]. Initially, IMH was considered as a distinct independent disease manifestation that arises from haemorrhage of the vasa vasorum of the aorta [18]. Increasingly, many authors have reported an overlap between classic AD and IMH [19, 20] and considered that the haematoma results from microscopic tears in the aortic intima [2]. Focal IMH has also been reported in association with PAU [20]. This widely propagated notion has resulted in a ‘classification’ of AAS that is used almost exclusively in the literature, where IMH is categorized as a separate ‘disease manifestation’ along with AD and PAU [1]. Adding to the confusion, the term IMH has been used inconsistently in the literature referring to a ‘disease’ equivalent, a variant of acute AD, and PAU.

Currently, it is accepted that IMH can be a variant or a precursor of AD, and many have regarded IMH as synonymous with a ‘thrombosed type’ or ‘non-communicating’ AD (Fig. 4) [15, 21] where separation of the medial layers still occurs, but this is filled with thrombus rather than flowing blood in what would otherwise be the false lumen of a classic dissection. On initial imaging, like at the follow-up imaging of IMH, small ‘ulcer-like projections’ (ULP)—defined as a localized blood-filled pouch protruding from the true lumen into the thrombosed false lumen of the aorta—can be observed [22]. In this context, the finding is considered to represent the site of an intimal disruption and is therefore a possible indicator of the formation of a flow channel between the true and thrombosed false lumen and hence evolves into classic AD. The term ‘ulcer-like lesion,’ however, is non-specific and has been used in the literature to include also non-penetrating as well as chronic ulcers of the aortic wall. In order to avoid confusion, we advocate using the term ‘primary intimal tear (PIT)’ for an intimal disruption in patients with the dissection variant IMH rather than using ‘ULP.’ The superior visualization of ECG-gated CTA dramatically improves the sensitivity for detecting small communications between the true and false lumen (PIT) in IMH (Fig. 4), which seems to be the elusive link between classic AD and IMH [6, 22]. In fact, a recent autopsy series showed that only 4 % of IMHs have no visible intimal tear; indeed, at the time of surgery a tear is found in most patients [7].

### Overlapping and transitional features of IMH

**Fig. 5:**
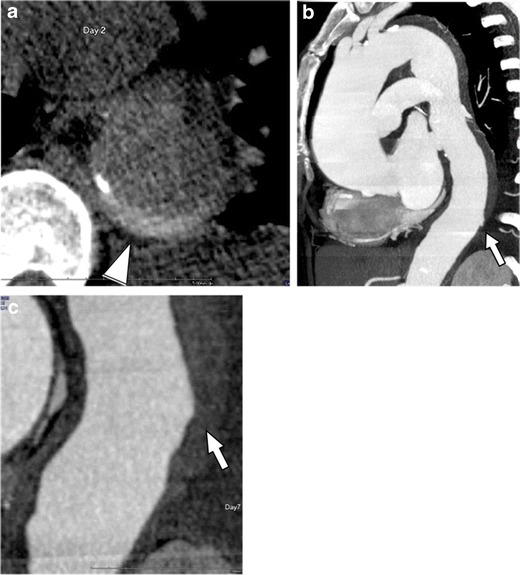
Resolved tiny primary intimal tear of dissection variant IMH in a 68-year-old woman with hypertension who presented with unstable thoracoabdominal pain. **a** Non-contrast CT image shows a crescent-shaped high-density area in the descending aorta (*arrowhead*). **b** Slab MIP image demonstrates haematoma extending through the whole thoracic descending aorta, which consists of dissection variant IMH. A tiny PIT is detected at the distal end of the haematoma (*arrow*). **c** The patient was followed with stable limited chest pain. After 7-day follow-up, the PIT resolved spontaneously, leaving a slightly enlarged haematoma (*arrow*). The patient was treated conservatively, and the haematoma eventually resolved

As we discussed above, it is well known that IMH may be a precursor of and evolve into classic dissection [13]. The prevalence of IMH in AAS has been reported to be 10 % to 50 %, and the prevalence in the recent studies reported less than previously thought [17, 23]. Many initially diagnosed IMHs may develop into classic AD [19, 24]. On the other hand, focal intimal injury (PIT) in IMH may heal and resolve (Fig. 5) [25]. Therefore, it is important to recognize that the imaging features may vary depending on the time period during the course of the disease as the pathological process underlying classic AD and IMH is intrinsically overlapped.

In addition to dissection variant IMH, a wide range of acute aortic lesions may demonstrate haemorrhagic content within the aortic wall to variable degrees, most importantly PAU, discussed below, but also iatrogenic ADs, traumatic injuries and rupturing aneurysms, etc. In this broad sense IMH can be regarded simply as a non-specific imaging finding, and in fact, cases that were previously categorized as IMH often included a spectrum of unrelated underlying aortic conditions. In order to avoid confusion and misunderstanding, we use the term ‘dissection variant IMH’ for a thrombosed AD that has no complete flow channel, but tiny communications between the true and false lumen commonly exist and clearly show differentiation from other aortic diseases with haemorrhagic content within the aortic wall.

## Limited intimal tear

### Definition

A limited intimal tear (also known as ‘incomplete tear’ or ‘subtle/discrete dissection’) is a rare variant of AD that is the least recognized disease manifestation of AAS [13, 14]. Pathologic descriptions of this type of lesion can be found as early as 1930 by Erheim [9], who used the term ‘nontraumatic laceration.’ Although the incidence of limited intimal tears is not well known and is likely underestimated because of general unfamiliarity with this dissection variant [14], the European Society of Cardiology included this lesion in its classification of dissection subtypes (subtle/discrete dissection, class 3) [21], which was reiterated in the guideline of the American College of Cardiology Foundation and the American Heart Association in 2010 [2]. The limited intimal tear is a subtype of AD that is characterized pathologically by a stellate or linear tear through the intima and underlying superficial media that results in exposure of the deeper media or adventitial layers [13]. The intimal tear extends to a variable degree within the aortic media without significant separation of the medial layers and does not result in a second flow channel, as seen in classic AD [13].

**Fig. 6:**
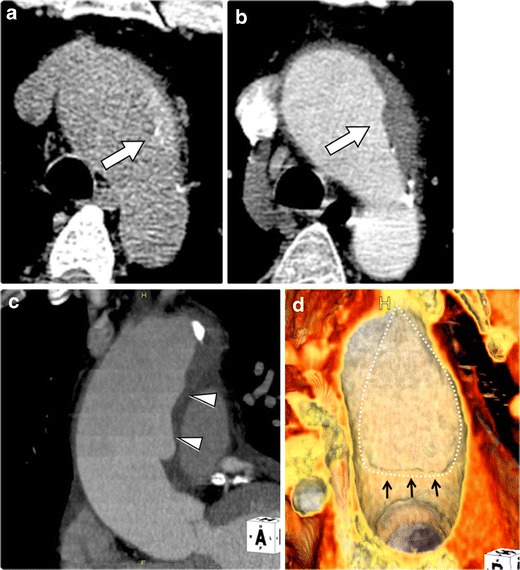
Limited intimal tear with intramural haemorrhagic content in a 48-year-old male. **a** Non-contrast CT demonstrates the aortic wall thickening with haemorrhagic content (*arrow*) at the proximal aortic arch. **b** Contrast-enhanced CTA demonstrates the aortic wall thickening (*arrow*) without clear intimal-medial flap or false lumen visualized; this was initially diagnosed as traditional IMH. **c** Slab-MIP image demonstrates the ‘eccentric one-sided bulge’ of the aortic wall along the ascending aorta (*arrowheads*). **d** 3D-VR luminal image right in front of the bulge shows a teardrop-shaped intimal tear (*dotted line*) with a localized intimal flap (*black arrows*) at the inferior border of the intimal tear

A limited intimal tear is particularly difficult to visualize with traditional imaging modalities [13]. The longitudinal tear on the intimal superficial surface is difficult to detect by most imaging modalities, although Chirillo et al. have reported the benefit of transesophageal echocardiography in detecting the intimal abnormality [14]. The eccentric bulge may be the only imaging finding of this lesion, which can be easily overlooked with traditional imaging modalities [13, 14]. Currently, ECG-gated CTA can have potential utility in detecting subtle forms of limited intimal tear [6]. In our experiences, limited intimal tear demonstrated a minor contour abnormality of the aortic wall (‘eccentric one-sided bulge’) on ECG-gated CTA (Fig. 6). Eccentric one-sided bulges are sometimes accompanied by haemorrhagic content within the aortic wall on non-contrast CT imaging and subtle undermined edges on CTA (Fig. 6). Advanced post-processing techniques—particularly 3D-VR and virtual luminal views—can greatly increase the conspicuity of these often extremely subtle limited intimal tears (Fig. 6) [6].

### Discriminating and overlapping features of limited intimal tear

Previously, limited intimal tears might have been diagnosed as other aortic diseases in AAS or missed all together because of the difficulty in detecting this discrete finding with standard imaging modalities [13, 26, 27]. As limited intimal tear remains an unfamiliar entity, the focal bulge associated with this lesion may be incorrectly diagnosed as either an atherosclerotic aneurysm or pseudoaneurysm. As a limited intimal tear and classic AD share the same pathophysiologic process—a weakened medial layer of the aortic wall—these two dissection subtypes may, not unexpectedly, show overlapping features. We have experienced some cases where an initial focal intimal tear evolves into a classic AD locally to form true and false lumens (Fig. 6). Overlapping imaging of features of IMH can also occur as limited intimal tears may also demonstrate a small amount of haemorrhagic content surrounding the tear within the aortic media (Fig. 6) [28].

## Penetrating atherosclerotic ulcer (PAU)

PAU is defined as an atherosclerotic ulceration that penetrates from the pathologically thickened intima through the internal elastic lamina into the media of the aortic wall [12]. PAU is a manifestation of advanced atherosclerosis and therefore a manifestation of a diseased intima (and not the media, as in AD) [12]. The lesion may penetrate even beyond the media and extend through to the adventitia, producing a periaortic pseudoaneurysm and even transmural aortic rupture [12].

It is important to appreciate the fundamental distinction in the underlying pathological processes between PAU and AD [29]. PAU occurs in the setting of severely and extensively diseased intima in patients with advanced atherosclerosis. Clinically, the typical profile of a patient with PAU is an elderly individual with multiple risk factors for atherosclerosis and often already documented manifestation of atherosclerotic disease, such as coronary aortic disease, cardiovascular disease, peripheral arterial disease or abdominal aortic aneurysm [13]. Patients with AD, on the other hand, are relatively younger and besides hypertension may not have any other risk factors for atherosclerosis [10, 12, 30].

Differentiation between PAU and a dissection variant IMH is clinically important as the prognosis and management differ depending on the underlying disease entity. PAUs arise in segments of the aorta where atherosclerotic changes are more common, and therefore over 90 % of PAUs are localized in the descending thoracic aorta [31]. Nevertheless, irrespective of their location, PAUs tend to have a bad prognosis—even when disease is limited to the descending aorta—with a higher incidence of aortic rupture compared with aortic dissection. For this reason, some authors advocate a more aggressive approach with endovascular treatment for acutely symptomatic PAUs in the descending aorta, as an open surgical approach is generally prohibitively risky in these patients with multiple comorbidities [16]. This is in contrast to acute type B dissection variant IMH or AD, which tends to be more stable and respond well to aggressive medical management. The less common PAU in the ascending aorta should also be managed surgically, like any acute type A aortic lesion.

### Discriminating and overlapping features of PAU

**Fig. 7:**
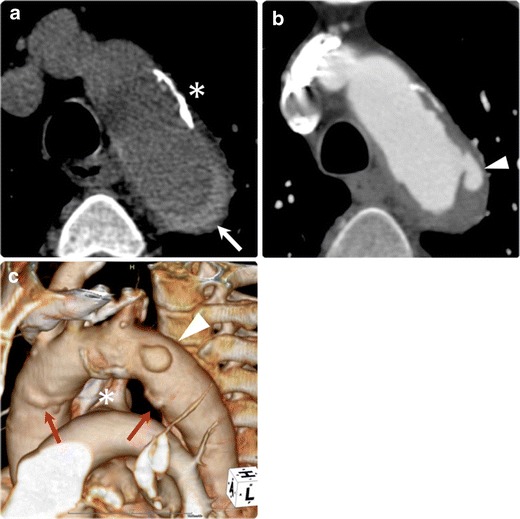
Penetrating atherosclerotic ulcer with haemorrhagic content in a 74-year-old man. **a** Non-contrast CT shows intramural haemorrhagic content (*arrow*) in the descending aorta. Note the thick calcification and plaque on the aortic wall (*), suggesting an atherosclerotic background of the patient. **b** Axial ECG-gated CTA demonstrates the ulcerative lesion at the proximal ascending aorta (*arrowhead*) into the aortic media, which shows associated haemorrhage. **c** 3D-VR image depicts the crater-like ulceration (*arrowhead*) and atherosclerotic calcification (*) on the aortic arch. Some non-penetrating atherosclerotic ulcers are also identified (*arrows*). The condition is considered as an overlapping feature between IMH (in the broad sense) and penetrating atherosclerotic ulcer

**Fig. 8:**
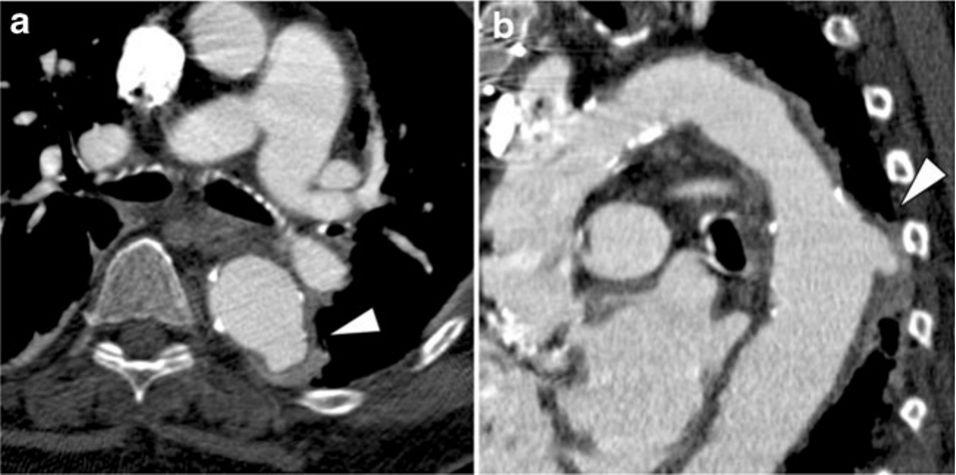
Penetrating atherosclerotic ulcer in an 81-year-old man. **a** Axial CTA images demonstrate an ulcerative lesion (*arrowhead*) on the thickened aortic wall with haemorrhagic content in the descending aorta. **b** Oblique sagittal MPR image clearly visualizes a crater-like feature of the ulcerative lesion (*arrowhead*) deeply penetrating into the atherosclerotic aortic wall. Entire aorta depicts severe atherosclerosis with plaque burden and calcifications

On imaging, patients with typical PAU invariably demonstrate extensive atheroma and calcification throughout the thoracic and abdominal aorta, often with an irregular surface (Figs. 7 and 8) [12]. Sundt et al. reported findings of PAU on transesophageal echocardiography, including a crater-like ulcer with jagged edges [29]. In our experience, PAUs commonly show a ‘crater-like ulceration’ of thickened aortic wall on ECG-gated CTA (Figs. 7 and 8), which is different from the linear or crescent shape of PIT in dissection variant IMH.

**Fig. 9:**
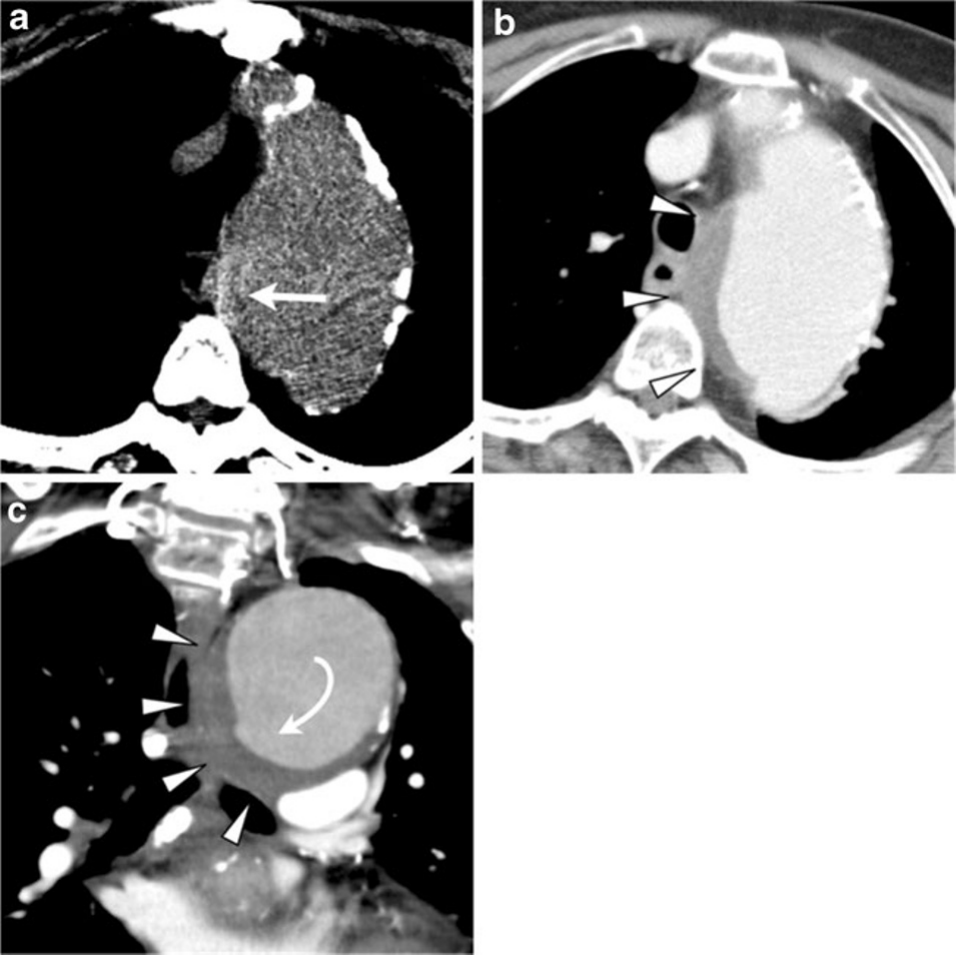
Impending rupture of an atherosclerotic aortic aneurysm in an 83-year-old man. The patient had been followed for stable aortic aneurysm for many years and suddenly presented with acute chest pain. **a** Non-contrast CT demonstrates ballooning of the aorta at the aortic arch. The aortic wall shows haemorrhagic content within the aortic wall (*arrow*), which suggests an acute process of this event. **b** Axial CTA depicts bulged dilatation of the aorta on the right side of the aortic arch (*arrowheads*), which was not depicted on the previous follow-up CT images. **c** Oblique coronal MPR image demonstrates dilatation of the aorta (*arrowheads*) with a thickened aortic wall with haemorrhagic content. The aortic lumen shows an eccentric bulge, a sign of impending rupture of an atherosclerotic aortic aneurysm

As the crater-like ulcerations of PAU are often accompanied by haemorrhagic content within the medial layer of the aortic wall [16], non-contrast enhanced CT demonstrates high-density haematoma in the aortic wall surrounding the ulceration (Fig. 9). Because of this accompanying haematoma, in the past, PAU has often been lumped together as ‘IMH’ with thrombosed AD (dissection variant IMH) as a cause of AAS [29]. Also, such lesions may have been diagnosed as ‘traditional IMH’ when ulceration was not evident enough in traditional imaging modalities, which caused the etiological confusion and misunderstanding with classic aortic dissection and its variants [16]. As discussed above, IMH is now more appropriately regarded as a variant of AD [15, 21], and its pathogenesis is different from that of PAU. In order to avoid confusion, we recommend that ‘PAU with haemorrhagic content’ that has an atherosclerotic pathogenesis should be clearly differentiated from the dissection variant IMH. The key to the differential diagnosis is underlying systemic atherosclerosis such as severe calcification of systemic arteries, coexisting cardiovascular diseases, aortoiliac and peripheral arterial occlusive diseases.

Nevertheless, clinical and imaging features of AD and PAU can overlap, especially in older patients, who often have two co-existing pathologic processes (i.e., severe atheroslceorsis, as well as ‘cystic media necrosis,’ which is also seen as part of normal aging). Elderly patients with many comorbidities may possess risk factors that indicate a predisposition to both atherosclerosis and AD simultaneously. In such patients, the differentiation between a limited intimal tear in AD and small atherosclerotic ulcer is sometimes difficult or impossible. In any case, the presence of acute haemorrhage in the aortic wall represents an aortic emergency irrespective of etiology.

## Impending rupture of aortic aneurysms

Thoracic aortic aneurysms (TAA) are often clinically asymptomatic and gradually increase in size over time. Although relatively uncommon, TAAs may rapidly increase in size, with increased risk of rupture (Fig. 9). The risk of rupture is closely associated with aneurysm size: for aneurysms greater than 6 cm in diameter, the risk of rupture is significantly higher [32]. Elective repair is indicated to prevent rupture. Rupturing TAAs may present as AAS with acute chest pain [33]. Again, the clinical presentation is indistinguishable from AD or PAU [33]. As such, although not included in the original classification of AAS by Vilacosta [1], we consider rupturing thoracic aortic aneurysms as one of the entities of AAS.

DeBakey originally used the term ‘dissecting aneurysm’ in 1965 for classic AD [34], which semantically put ‘aneurysm’ and ‘dissection’ into one disease category. While our understanding of AAS has evolved over the last 50 years, some confusion with respect to those two pathologic conditions still remains. A true aortic aneurysm is defined as dilatation of the aorta that contains all layers of the aortic wall and usually involves the entire circumference [33]. Pathologically, an aneurysm is characterized by deterioration of the aortic wall with loss of elastin and smooth muscle cells [33]. Reed et al. reported the clear etiological difference between degenerative aneurysm and dissection [35]. The most striking difference was the age-specific incidence rates: aortic aneurysm increased steadily with 50 years of age, whereas AD peaked around 65 years and decreased thereafter [35]. Degenerative aneurysms are also considered as a manifestation of atherosclerosis.

### Atherosclerotic aneurysm vs. aneurysmal dilatation of various aortic pathologies

**Fig. 10:**
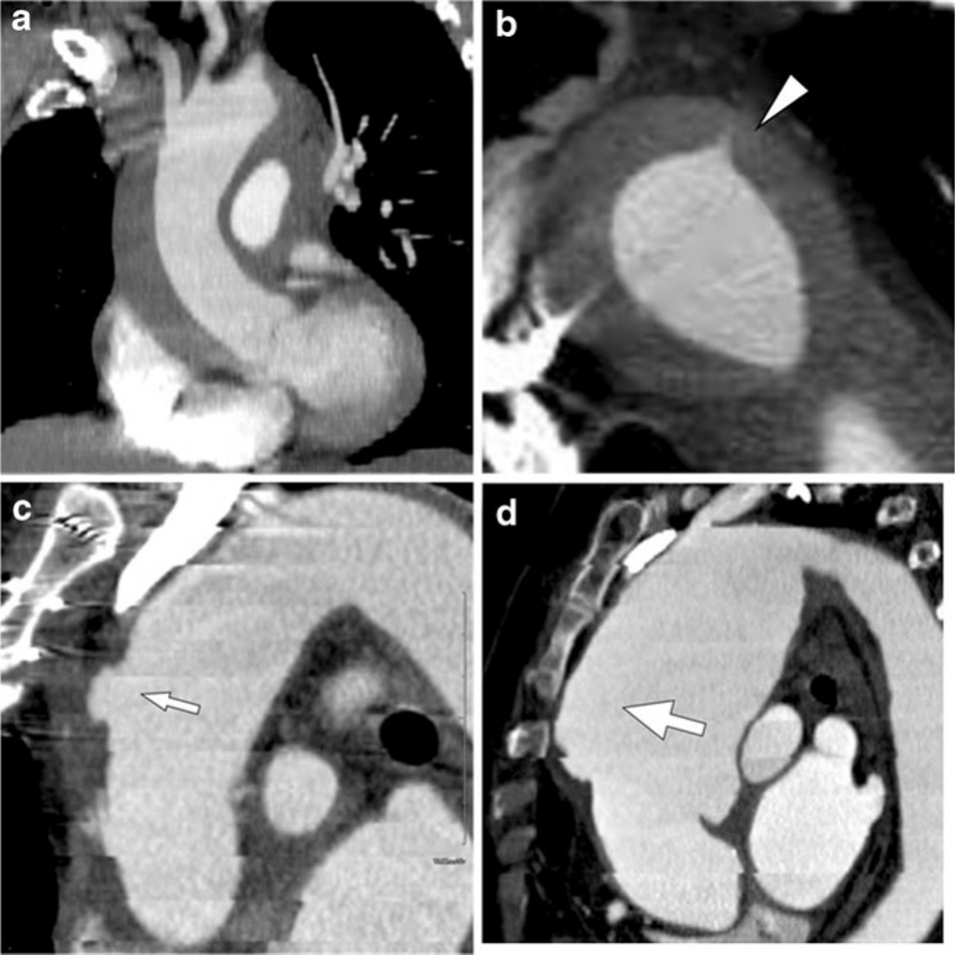
Aneurysmal dilatation of the aorta secondary to dissection variant IMH in a 66-year-old woman. **a** Oblique sagittal contrast-enhanced CT demonstrates IMH extending from the ascending aorta through the proximal descending aorta. **b** Axial image depicts a streak-form ulcer-like projection (primary intimal tear) in the descending aorta (*arrowhead*). **c** Two days after onset, an ulcer-like projection was enlarged to form an outpouching cavity into the thrombosed false lumen (*small arrow*). **d** One week later, the haemorrhagic content is resolved and the ulcer-like projection not been detected. On the other hand, the aorta shows aneurysmal dilatation. Note that the dilatation starts from the location where the haematoma was present (*large arrow*). Although the pathological features and clinical course are different, such aneurysmal dilatation of dissection variant IMH might be confused with an atherosclerotic aortic aneurysm if an ulcer-like projection is not detected on the initial CT examination

In addition to degenerative aneurysms, many kinds of aortic pathology may show aneurysmal dilatation in their chronic stage. Chronic AD with associated aneurysmal dilatation of the false lumen can be easily differentiated from standard atherosclerotic aortic aneurysms, even if the intimomedial flap is chronically degenerated, fragmented and partially absent. However, if a dissection variant IMH shows dilatation of the aorta, this may not always be easily differentiated from a true aortic aneurysm, and once the haemorrhagic content is resolved in the subacute and chronic phase, these lesions may be indistinuishable (Fig. 10). Limited intimal tears can heal and may appear as an eccentric aneurysm in the chronic phase and continue to dilate over time (Fig. 6). Chronic PAU may also show aneurysmal dilatation by ‘aortic remodeling,’ which is usually focal and eccentric [31].

## Conclusion

The conceptual classification of culprit aortic lesions of AAS into pathologies involving either a diseased media (medial degeneration) or a diseased intima (advanced atherosclerosis) avoids some of the confusing terminology regarding AAS. In addition to the discriminating features of each aortic disease, recognition of the overlapping and transitional features in the dynamic and evolving process of AAS will allow a more comprehensive understanding of their underlying pathophysiologic conditions and their natural history, and may improve strategies for therapeutic management. Understanding these features will provide the radiologist with helpful clues facilitating image assessment, allowing further prediction of the natural disease course as well as permitting appropriate management recommendations.
